# Pheochromocytoma Presenting With Paroxysmal Hypertensive Crises in a Previously Healthy Woman: A Case Report

**DOI:** 10.7759/cureus.100754

**Published:** 2026-01-04

**Authors:** Sona Maghakyan, Andranik Aleksanyan

**Affiliations:** 1 General Medicine, Mikaelyan University Hospital, Yerevan, ARM; 2 General Surgery, Mikaelyan University Hospital, Yerevan, ARM

**Keywords:** adrenal tumor, catecholamine-secreting tumor, hypertensive crisis, laparoscopic adrenalectomy, neuroendocrine tumor, paroxysmal hypertension, pheochromocytoma, secondary hypertension

## Abstract

Pheochromocytomas are rare catecholamine-secreting neuroendocrine tumors that typically present with episodic headaches, sweating, palpitations, and hypertension. Delayed diagnosis, although uncommon, can lead to severe cardiovascular complications.

We report the case of a 41-year-old woman with no known comorbidities who presented with a 20-day history of recurrent episodes of severe hypertension, with blood pressure reaching 220/110 mmHg, accompanied by headaches, palpitations, tremor, and diaphoresis. Biochemical evaluation revealed elevated urinary metanephrines and normetanephrines. Abdominal computed tomography (CT) demonstrated a 6-cm left adrenal mass.

After appropriate preoperative alpha- and beta-adrenergic blockade, the patient underwent a successful laparoscopic left adrenalectomy. Histopathology confirmed pheochromocytoma. Postoperatively, her blood pressure normalized, antihypertensive therapy was discontinued, and she was advised to undergo genetic testing and regular follow-up.

Early recognition of pheochromocytoma is essential, as delayed diagnosis can result in severe and potentially life-threatening complications. This case emphasizes the importance of considering pheochromocytoma in middle-aged patients presenting with unexplained paroxysmal hypertension and adrenergic symptoms, as timely surgical management can be curative.

## Introduction

Pheochromocytomas are rare catecholamine-producing neuroendocrine tumors arising from chromaffin cells of the adrenal medulla [1]. These tumors secrete excessive catecholamines, including epinephrine, norepinephrine, and dopamine, leading to episodic or sustained sympathetic hyperactivity. The estimated annual incidence is approximately 0.8 per 100,000 person-years [2], and pheochromocytomas account for fewer than 1% of hypertension cases. However, they represent a clinically important and potentially curable cause of secondary hypertension.

Excess catecholamine release results in vasoconstriction, tachycardia, and metabolic disturbances, explaining the classic triad of episodic headaches, diaphoresis, and palpitations, often accompanied by paroxysmal or sustained hypertension [3]. The clinical presentation is highly variable, and symptoms may be intermittent, which contributes to delayed diagnosis or misdiagnosis [4]. If unrecognized, pheochromocytoma may lead to severe cardiovascular complications, including arrhythmias, cardiomyopathy, myocardial infarction, and stroke.

Advances in genetic testing have demonstrated that a substantial proportion of pheochromocytomas are associated with germline mutations, even in patients without a family history; current guidelines recommend genetic evaluation for all affected individuals [5].

We present a case of pheochromocytoma in a previously healthy middle-aged woman who presented with recurrent hypertensive crises, highlighting the importance of early recognition, careful preoperative management, and definitive surgical treatment.

## Case presentation

A 41-year-old woman with no known comorbidities presented with a 20-day history of recurrent severe hypertension, with blood pressure readings up to 220/110 mmHg, accompanied by headaches, palpitations, tremor, and profuse sweating. Physical examination revealed pallor but was otherwise unremarkable.

Electrocardiography showed sinus tachycardia at 140 beats per minute, incomplete right bundle branch block, and nonspecific T-wave changes. Troponin I was within normal limits (5.78 pg/mL; reference range 0-20 pg/mL), and transthoracic echocardiography revealed no significant abnormalities.

Laboratory evaluation demonstrated elevated 24-hour urinary metanephrines (493 µg/24 h; reference <312 µg/24 h) and normetanephrines (515 µg/24 h; reference <445 µg/24 h) (Table 1).

**Table 1:** Laboratory findings at presentation

Laboratory Test	Patient Value	Reference Range
24-hour urinary metanephrines	493 µg/24 h	<312 µg/24 h
24-hour urinary normetanephrines	515 µg/24 h	<445 µg/24 h
Troponin I	5.78 pg/mL	0-20 pg/mL

**Figure 1:**
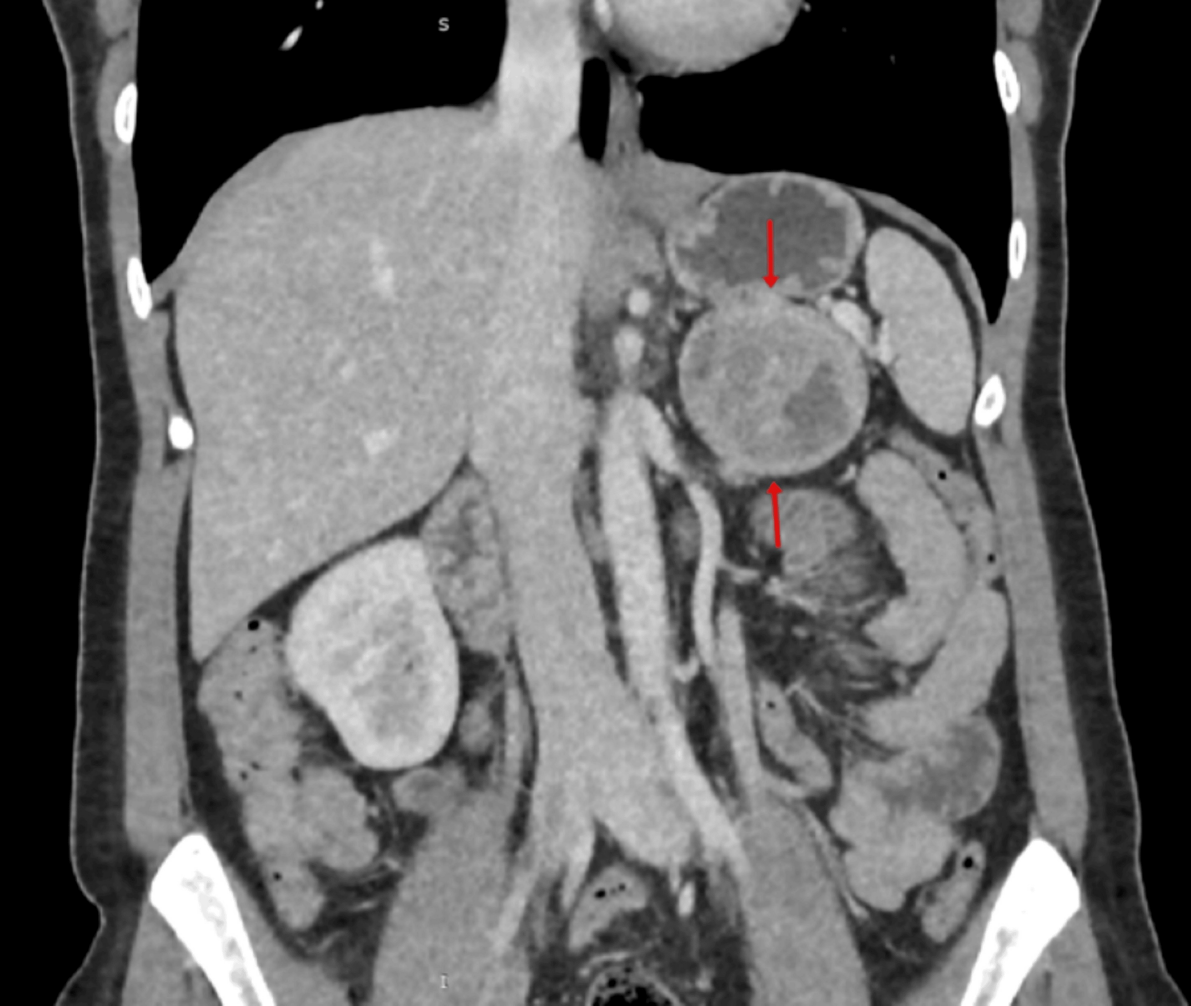
Computed tomography (CT) scan showing a left adrenal mass (arrows)

Abdominal computed tomography (CT) identified a 6-cm mass in the left adrenal gland without evidence of metastatic disease (Figure 1).

A diagnosis of pheochromocytoma was established. Preoperative medical management with alpha-adrenergic blockade, followed by beta-adrenergic blockade, was initiated to achieve adequate blood pressure control. After 10 days of optimization, the patient underwent a successful laparoscopic left adrenalectomy. Histopathology confirmed pheochromocytoma. Postoperatively, her blood pressure and blood glucose levels normalized, antihypertensive medications were discontinued, and she was advised to undergo genetic testing and regular outpatient follow-up.

## Discussion

Pheochromocytoma is a rare but clinically significant cause of secondary hypertension due to excessive catecholamine secretion, which may result in severe cardiovascular complications if unrecognized. Although it accounts for fewer than 1% of all hypertension cases, its prevalence among patients evaluated for secondary hypertension is higher, emphasizing the need for clinical vigilance [6]. The episodic and nonspecific nature of symptoms frequently leads to diagnostic delay, particularly in patients without a prior history of hypertension.

Paroxysmal hypertensive crises, as observed in this case, are characteristic but not universal. These episodes are often accompanied by adrenergic symptoms such as headaches, palpitations, tremor, diaphoresis, and tachycardia [4]. Cardiovascular manifestations may range from transient electrocardiogram (ECG) changes to arrhythmias, catecholamine-induced cardiomyopathy, myocardial infarction, and stroke [7]. Early recognition is essential to prevent irreversible organ damage.

Paroxysmal hypertension with adrenergic symptoms poses a diagnostic challenge, as several conditions may mimic pheochromocytoma. Differential diagnoses include panic disorder, thyrotoxicosis, carcinoid syndrome, obstructive sleep apnea, and drug-induced sympathetic activation. These conditions may present with episodic palpitations, diaphoresis, and elevated blood pressure, but are typically distinguished by clinical context, laboratory evaluation, and the absence of markedly elevated catecholamine metabolites. In this case, recurrent hypertensive crises combined with elevated urinary metanephrines and normetanephrines strongly supported the diagnosis of pheochromocytoma, which was subsequently confirmed by imaging and histopathology. Early biochemical evaluation is therefore crucial in differentiating pheochromocytoma from more common causes of episodic adrenergic symptoms.

Biochemical confirmation using plasma or urinary fractionated metanephrines remains the most sensitive diagnostic approach [3,8]. Once biochemical evidence is obtained, imaging studies such as CT or magnetic resonance imaging are used to localize the tumor [9]. Surgical resection is the definitive treatment; however, appropriate preoperative preparation with alpha-adrenergic blockade, followed by beta-adrenergic blockade when indicated, is mandatory to minimize perioperative risk [10,11]. Minimally invasive adrenalectomy is the preferred approach for localized adrenal tumors and is associated with excellent outcomes [12].

Given the risk of recurrence and the increasing recognition of hereditary pheochromocytoma, long-term follow-up and genetic evaluation are recommended for all patients [5]. This case highlights the importance of maintaining a high index of suspicion for pheochromocytoma in patients presenting with unexplained paroxysmal hypertension and adrenergic symptoms, as timely diagnosis and appropriate surgical management can be curative.

## Conclusions

Pheochromocytoma is a rare but potentially life-threatening cause of hypertension. It should be considered in middle-aged patients presenting with unexplained paroxysmal hypertension and adrenergic symptoms. Early diagnosis, appropriate preoperative preparation, and surgical resection can resolve symptoms and prevent serious complications.
